# A microchip platform for structural oncology applications

**DOI:** 10.1038/npjbcancer.2016.16

**Published:** 2016-06-15

**Authors:** Carly E Winton, Brian L Gilmore, Andrew C Demmert, Vasilea Karageorge, Zhi Sheng, Deborah F Kelly

**Affiliations:** 1 Virginia Tech Carilion Research Institute, Roanoke, VA, USA; 2 School of Biomedical Engineering and Science, Virginia Tech, Blacksburg, VA, USA; 3 Virginia Tech Carilion School of Medicine, Roanoke, VA, USA; 4 Department of Biological Sciences, Virginia Tech, Blacksburg, VA, USA

## Abstract

Recent advances in the development of functional materials offer new tools to dissect human health and disease mechanisms. The use of tunable surfaces is especially appealing as substrates can be tailored to fit applications involving specific cell types or tissues. Here we use tunable materials to facilitate the three-dimensional (3D) analysis of BRCA1 gene regulatory complexes derived from human cancer cells. We employed a recently developed microchip platform to isolate BRCA1 protein assemblies natively formed in breast cancer cells with and without BRCA1 mutations. The captured assemblies proved amenable to cryo-electron microscopy (EM) imaging and downstream computational analysis. Resulting 3D structures reveal the manner in which wild-type BRCA1 engages the RNA polymerase II (RNAP II) core complex that contained K63-linked ubiquitin moieties—a putative signal for DNA repair. Importantly, we also determined that molecular assemblies harboring the *BRCA1*^*5382insC*^ mutation exhibited altered protein interactions and ubiquitination patterns compared to wild-type complexes. Overall, our analyses proved optimal for developing new structural oncology applications involving patient-derived cancer cells, while expanding our knowledge of BRCA1’s role in gene regulatory events.

## Introduction

Mutations in the breast cancer susceptibility protein (BRCA1) are known to contribute to cancer induction.^1,2^ At the molecular level, the intricate details of these events are poorly understood. During normal cellular activities, BRCA1 interacts with its binding partner, BARD1 (BRCA1-associated ring domain protein), to ensure genomic stability and cell survival.^3^ In this context, BRCA1 functions as a tumor suppressor by safeguarding genetic material.^4–6^ A critical opportunity to monitor for errors in DNA, and to correct them, occurs during RNA synthesis. The BRCA1–BARD1 heterodimer has an important role in this process as BRCA1-related repair proteins are found in proximity to exposed DNA during transcription.^7,8^ However, the precise manner in which BRCA1 works in concert with RNA polymerase II (RNAP II) is ill-defined.

Currently, there is little structural information available for BRCA1 protein assemblies, despite their well-known contribution to human disease. This lack of information is due to many factors including: (1) the size of the BRCA1 protein (~208 kDa) makes it difficult to express recombinantly; (2) the inherent flexibility of full-length BRCA1 renders it problematic to crystallize; and (3) few strategies are available to isolate BRCA1 protein assemblies from human tumor cells for structural analysis. The size and flexibility of BRCA1 are intrinsic properties of the protein that shape its biological activity, and are thus not easy to modify in patient-derived cell lines.

As an alternative strategy we chose to develop new tools to investigate protein complexes naturally formed in human breast cancer cells. Specifically, we have recently reported the production of the tunable microchip system, which enabled the first structural analysis of BRCA1 protein assemblies.^9^ As part of our work to establish the microchip system, we determined a likely scenario to explain how BRCA1 associates with the RNAP II core complex. We resolved the position of the BRCA1 C-terminal domain (BRCT) with respect to the RNAP II core, and distinguished the level of structural variability present in the biological samples. Information that was missing from these initial analyses, however, included a more detailed understanding of the BRCA1 N-terminal (RING) domain, and the manner in which ubiquitin patterns affect protein–protein interactions.

Here we present biochemical and structural results that expand upon these initial findings and reveal new molecular insights for BRCA1 protein architectures. These results show the proximity of the BRCA1 RING domain in relation to DNA fragments that were bound to transcriptional assemblies. We also define regions on the RNAP II core that accommodate K63-linked ubiquitin moieties, which are known signals for DNA repair mechanisms. Equally importantly, we now illustrate that the 3D structures of wild-type and mutated BRCA1 assemblies vary considerably. Taken together, our technical advances provide a new molecular framework to study gene regulatory assemblies with and without cancer-related mutations. As such, we refer to this exciting new opportunity as ‘structural oncology.’

## Results

### Capturing BRCA1 complexes from breast cancer cells for structural analysis

**Figure 1:**
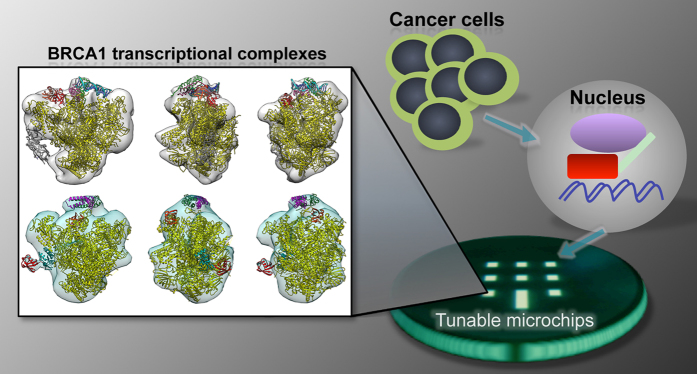
The tunable microchip system captures native proteins produced in breast cancer cells. Native BRCA1 protein assemblies formed in the nucleus of hereditary breast cancer cells were tethered to tunable SiN-based microchips for 3D structural analysis. Representative 3D reconstructions (white and cyan) show variations in structural features and molecular domains as described in the present work.

We recently established a streamlined approach to isolate native BRCA1 assemblies from the nuclear contents of primary ductal carcinoma cells (HCC70 line).^9^ Here we employed the same strategy to examine new molecular interfaces of wild-type assemblies, and to compare how these interfaces differ among mutated complexes (summarized in Figure 1). Briefly, RNAP II, BRCA1, and BARD1 contained in the nuclear material of HCC70 cells were naturally enriched and co-eluted from Nickel–Nitrilotriacetic acid (Ni–NTA) agarose beads. In the eluted fractions we found that wild-type BRCA1 associated with BARD1 and the RNAP II large subunit (RPB1) as determined by co-immunoprecipitation (co-IP) experiments. In addition, the RNAP II complexes were post-translationally phosphorylated at pSer5/pSer2 peptide repeats, and ubiquitinated with K63-type linkages (Supplementary Figure S1). After verifying these biochemical associations, we used the microchip system to examine the molecular arrangements of the proteins that constituted the BRCA1 assemblies.

### 3D structures of wild-type BRCA1 assemblies reveal new molecular interfaces

To gain structural insights of BRCA1–RNAP II interactions, we applied aliquots of the eluted fractions to Cryo-SiN microchips^10^ decorated with antibodies against either the structured BRCA1 N- or C-terminal domains. This step selected for BRCA1-associated RNAP II complexes and excluded those complexes not bound to BRCA1, as previously described.^9^ Tethered protein assemblies were then plunge-frozen into liquid ethane for cryo-EM imaging and downstream analysis (see Materials and Methods section for a full description of imaging procedures).

**Figure 2:**
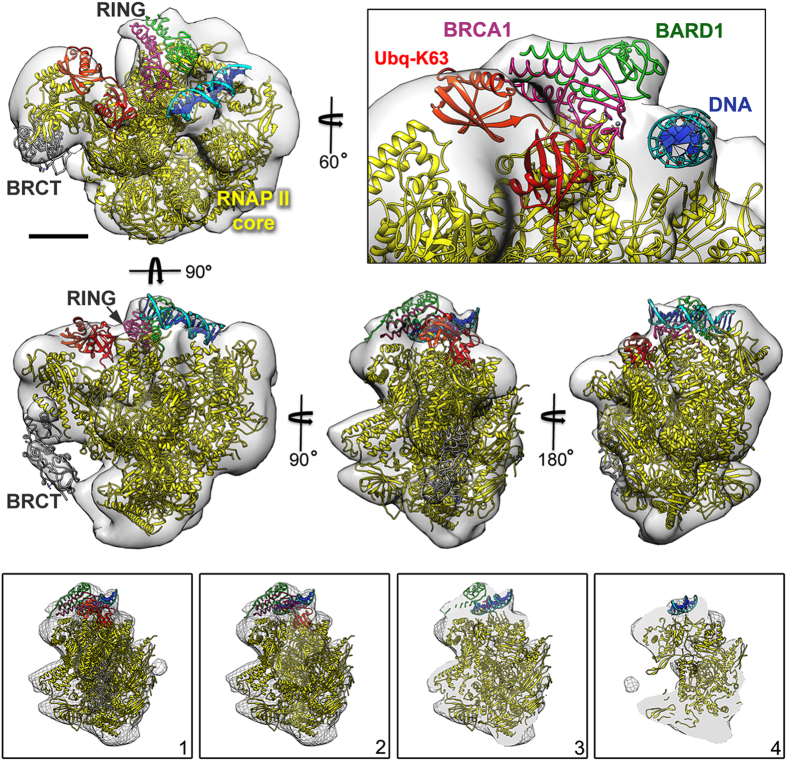
EM structure reveals BRCA1 domains directly engage the RNAP II core proximal to DNA in human breast cancer cells. The EM density map (white) is shown in different orientations. The position of the BRCT domain^13^ was recently determined based on antibody-labeling results.^9^ In the present study, the BRCA1–BARD1 RING domain^6^ was uniquely placed into the density map. The DNA strand (blue) was positioned over the DNA channel accordingly.^15^ K63-linked ubiquitins^17^ occupied the remaining density. The RNAP II core was localized in the EM map based on a model of the RNAP II X-ray crystal structure.^18^ Bar = 5 nm. Cross-sections through the density map (1–4) indicate the overall fit of the atomic models within the envelope. Also see Supplementary Figure S3 and Supplementary Movie S1. BRCT, BRCA1 C-terminal domain; BARD1, BRCA1-associated ring domain protein; EM, electron microscopy; RNAP II, RNA polymerase II.

We employed computational procedures^11,12^ to separately determine the positions of where the BRCA1 structural domains interacted with the RNAP II assemblies, based on antibody-labeling results. The extra major densities found in the experimentally determined EM density map were attributed to either the BRCA1–BARD1 N-terminal RING domains or the C-terminal BRCT domain (Figure 2). The orientation of the BRCT domain (pdbcode, 1JNX)^13^ was previously resolved and found to be proximal to the C-terminal region of the RNAP II core.^9^ This observation is consistent with other biochemical findings.^7,14^

Antibody-labeling results also indicated the unoccupied density in the EM map located near the DNA channel was attributed to the BRCA1–BARD1 N-terminal RING domain. This information now permits us to place the structure of the RING domain into the density map, which fit uniquely within the 3D envelope (pdbcode, 1JM7)^6^ (Figure 2, magenta and green). We further assigned a minor density over the DNA channel to a short strand of DNA (Figure 2, blue). This position of the DNA strand is in the same location described in other models of RNAP II engaging DNA in a ‘closed state’ during the initial stages of transcription.^15,16^ Additional modeling experiments guided the placement of the K63-linked ubiquitin moieties (pdbcode, 1UBQ)^17^ (Figure 2; orange and red). The current position of the ubiquitin moieties did not introduce atomic clashes. Other minor differences between the fit crystal structures and the experimentally determined 22-Å density map (0.5 FSC criteria; Supplementary Figures S2 and S3; Supplementary Movie S1) may be attributed to missing loops in the yeast RNAP II crystal structure (pdbcode, 4A93),^18^ and the fact that the complexes in our investigation were derived from human tumor cells, rather than from yeast. Similar differences were also noted in a previously determined EM structure derived from other immortalized human cell lines.^19^ Moreover, as the central region of BRCA1 is highly flexible, it is reasonable that we cannot fully visualize this region of the protein in the reconstruction. Collectively, these structural results indicated that phosphorylated RNAP II core complexes (1) interacted with BRCA1 N- and C-terminal domains, (2) contained K63-linked ubiquitin moieties, and (3) are likely primed for DNA repair. These findings were in good agreement with our biochemical assessments.

### BRCA1 directly engages DNA and the RNAP II core

**Figure 3:**
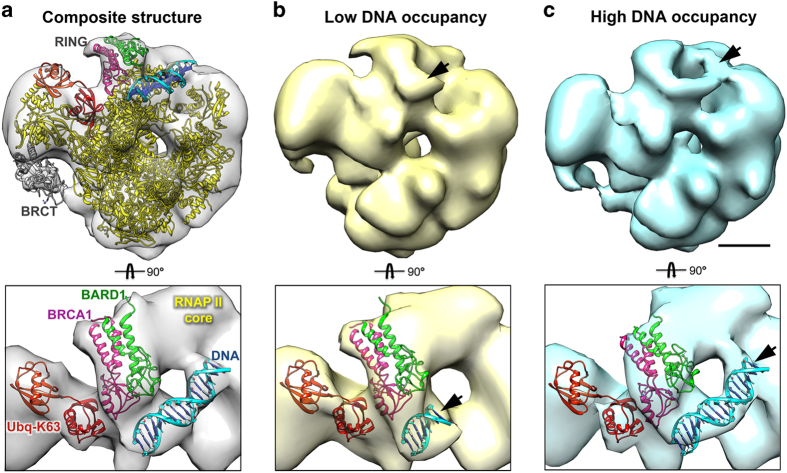
BRCA1 engages DNA in a variable manner while bound to the RNAP II core. (**a**) The composite EM structure was compared to transient intermediate structures having low and high DNA occupancies. (**b**) An intermediate structure having low DNA occupancy (yellow density map) accommodates a short fragment of DNA (blue) located proximal (black arrows) to the BRCA1–BARD1 RING domains.^6^ Limited density was present in the density map to accommodate K63-linked ubiquitins.^17^ (**c**) An intermediate structure having high DNA occupancy (cyan density map) accommodates a longer strand of DNA (blue) located proximal (black arrows) to the BRCA1–BARD1 RING domains. Bar=5 nm. Also see Supplementary Movie S2 and S3. BARD1, BRCA1-associated ring domain protein; EM, electron microscopy.

To test whether transient intermediate states were present in our samples, we utilized the RELION software package.^12^ Statistical output generated by RELION identified multiple structures were represented in the original image stack. The number of 3D classes determined by RELION was independent of user-defined starting parameters, and resulting 3D classes showed subtle variations in density (Figures 3a–c, black arrows). Comparing the composite EM map to the structures having the lowest and the highest DNA density, we noted potential differences in DNA engagement (Figure 3b,c; Supplementary Figures S4 and S5; Supplementary Movie S2 and S3). As these structures were determined from native assemblies, gently removed from the nuclear material, we posit that the observed heterogeneity may be due to differences in functional states. As such, we interpreted the low occupancy structure to represent a weakly bound DNA state, and the high occupancy structure to represent a strongly bound DNA state. Other important differences noted in the high occupancy structure included greater density for the K63-linked ubiquitins and for the BRCT domain (Figure 3; red, orange, and gray). These findings are consistent with BRCA1 complexes engaging DNA through a series of concerted steps that may be linked to DNA repair. Similar observations have been described in functional studies.^8,14,20,21^

### The BRCT domain prefers specific phospho-peptide sequences

**Figure 4:**
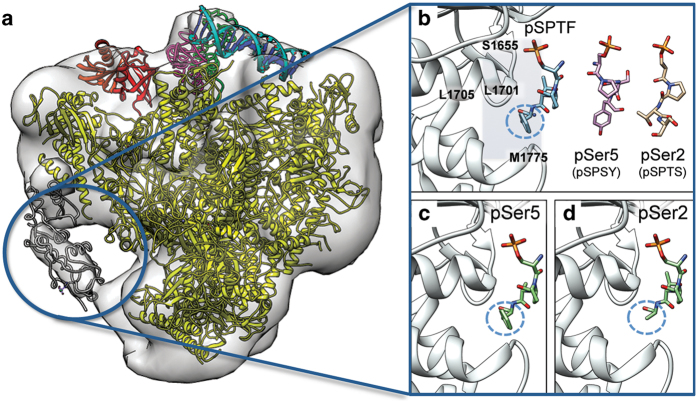
The pSer5 peptide exhibits the optimal stereochemistry to interact with the BRCT domain. (**a**) The composite 3D structure highlighting the BRCT domain (gray) within the density map. (**b**) A close-up view of the BRCT crystal structure (pdbcode, 3K0H ^22^) showing that the hydrophobic binding pocket (gray rectangle) accommodates a known peptide, pSPTF. Molecular modeling experiments were performed to overlay the pSer5 and pSer2 peptides onto the pSPTF model. (**c**) The pSer5 peptide contains a terminal tyrosine residue (blue dashed circle) that fits within the hydrophobic binding cleft. Also see Supplementary Movie S4. (**d**) The pSer2 peptide contains a terminal serine residue (blue dashed circle) that does not maintain the proper stereochemistry to optimally fit within the BRCT binding site. BRCT, BRCA1 C-terminal domain.

In addition to the variability noted near the DNA-binding site, we also found differences in the region containing the BRCT domain. As previously noted, the BRCT domain is adjacent to the C-terminus of the RNAP II core (Figure 4a). This region of RNAP II emanates from residue P1455 but is disordered in the crystal structure.^18^ As this disordered region is highly mobile, it can conceivably interact with the BRCT domain. To provide a conceptual framework for this interaction, we examined the substrate peptide pSPTF (Figure 4b) that was co-crystalized with the BRCT domain (pdbcode, 3K0H).^22^ Phosphorylated peptides are highly repetitive in the RNAP II C-terminus, and include the pSer5 (pSPSY) and pSer2 (pSPTS) consensus sequences. Atomic models for the pSer5 and pSer2 peptides have also been independently crystallized (pdbcode, 4H3K).^23^

In the course of the present study, we performed molecular modeling experiments to test for optimal BRCT–peptide interactions. We overlaid the model for the pSer5 peptide onto the substrate peptide that was co-crystalized within the BRCT domain. The pSer5 peptide model fit within the BRCT binding cleft that is defined by residues S1655, L1701, L1705, and M1775 (Figure 4b,c; Supplementary Movie S4). However, the pSer5 peptide contained a unique tyrosine residue in the consensus sequence compared with the analogously positioned phenylalanine residue. Although mutagenesis studies have reported that phenylalanine is the preferred residue that fits within the BRCT domain,^24,25^ our modeling results suggest that the heptad repeats in the RNAP II C-terminal domain may also have binding potential for this region. In particular, the pSer5 peptide more closely matches the stereochemistry requirements of the BRCT binding cleft than the pSer2 peptide (Figure 4d), which contains a terminal serine residue and is not likely to fit. These interactions are important to further investigate as many mutations in the BRCT are implicated in cancer induction. Therefore, we examined BRCA1 assemblies in cancer cell lines having a naturally mutated BRCT domain and known deficiencies in transcriptional activities.

### Differences exist between the wild type and mutant BRCA1 protein complexes

To examine the molecular architecture of mutated BRCA1-transcriptional assemblies, we implemented the same biochemical and structural approaches described for the wild-type complexes. We probed the nuclear material of breast cancer cells (HCC1937 line) that harbors a homozygous BRCA1 mutation (BRCA1^5382insC^). This mutation in the *BRCA1* gene is associated with deficiencies in transcription-coupled repair events and high incidences of breast cancer.^1,2^

We hypothesized that the functional differences observed in cells containing mutated BRCA1^5382insC^ may be related to its ability to form proper protein assemblies. To test this idea, we used the tunable microchip platform to isolate transcriptional assemblies containing BRCA1^5382insC^. We collected transmission electron microscopic (TEM) images of the mutated protein assemblies under the same low-dose conditions used for wild-type complexes. Individual assemblies were selected from the images using the PARTICLE program,^11^ and exported to the RELION software package.^12^ Implementing standard reconstruction procedures in RELION, we calculated the first 3D structure of mutated BRCA1^5382insC^ transcriptional assemblies.

**Figure 5:**
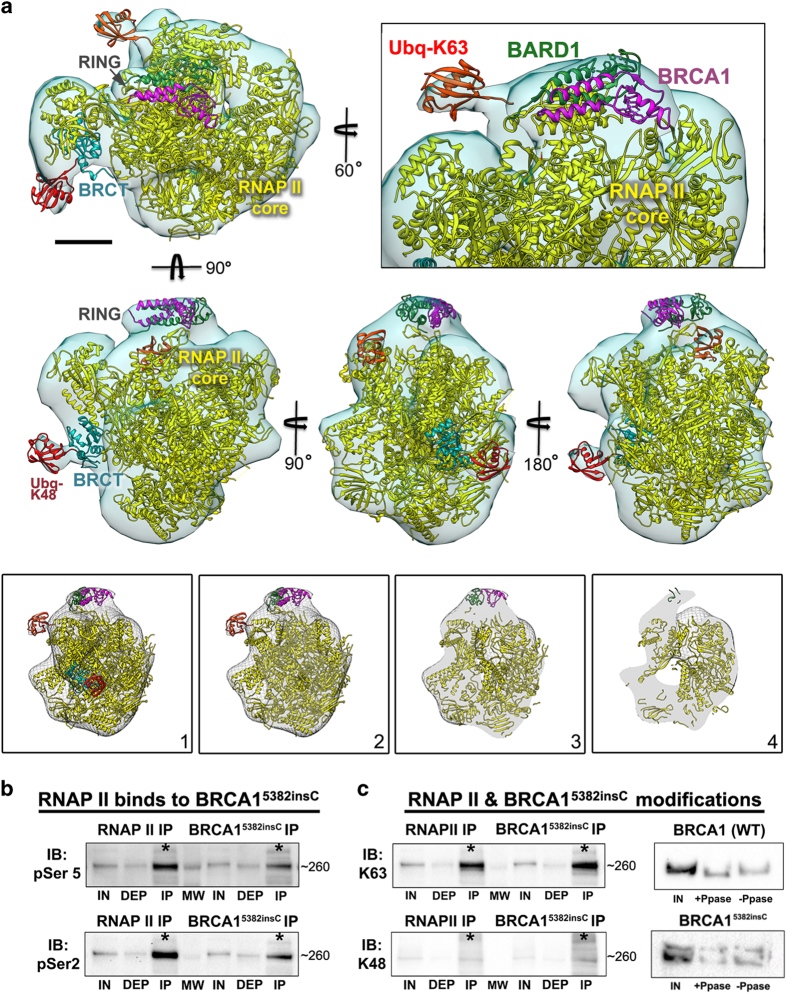
EM structure of mutated BRCA1^5382insC^ transcriptional complexes. (**a**) The EM density map (cyan) shown in different orientations was calculated using the RELION software package. Placement of the BRCA1–BARD1 (magenta, green) RING domains^6^ and the BRCT (aqua)^13^ varied compared with the wild-type structure. Models for ubiquitin modifications (red and orange)^17^ occupied the remaining minor density. RNAP II (yellow) was localized in the EM map based on a model of the RNAP II X-ray crystal structure.^18^ Bar = 5 nm. Additional cross-sections through the density map (1–4) indicate the fit of the atomic models within the envelope. Also see Supplementary Figure S7 and Supplementary Movie S5. (**b**) Western blot analysis of co-IP experiments showed the RNAP II core was phosphorylated at pSer5 and pSer2 peptide repeats, while interacting with mutated BRCA1^5382insC^. (**c**) The RNAP II core contained K63-linked ubiquitin moieties, while K48-type linkages are likely present on BRCA1^5382insC^. Wild-type BRCA1 shows a bandshift upon digestion with lambda phosphatase (+Ppase) in comparison with control samples lacking the enzyme (-Ppase). Mutated BRCA1^5382insC^ does not show a change in migration upon incubation with lambda phosphastase. * denotes protein interactions. DEP, unbound material; EM, electron microscopy; IB, immunoblot; IN, input material; IP, immunoprecipitated interaction; RNAP II, RNA polymerase II.

The EM density map of the mutated BRCA1 complex was interpreted by drawing from information made available from the wild-type structure. First, we positioned the RNAP II core (pdbcode, 4A93)^18^ and BRCA1–BARD1 RING domain (pdbcode, 1JM7)^6^ into the density map (Figure 5a; Supplementary Figures S6 and S7, Supplementary Movie S5). Upon comparing the mutant and wild-type structures, there was a notable difference in orientation of the BRCA1–BARD1 RING (Figure 5a, green and magenta) domain relative to the RNAP II core (Figure 5a, yellow). Another major difference we noted was that the full-length BRCT domain did not fit well in the map of the mutated complex. We reasoned that the limited density seen in the region of the mutated BRCT was due to the frameshift mutation that imparts a stop codon, resulting in a protein truncation.

Other studies have shown that truncations in the BRCT domain render it susceptible to proteolysis, whereas the full-length protein is highly resistant to cleavage.^26^ The fact that the mutated complexes were tethered to the microchips by polyclonal antibodies against the BRCT, indicate that some portion of this domain is intact and properly folded. Therefore, we placed a homology model of the truncated BRCA1^5382insC^ domain (Figure 5a, aqua) into the density available in this region of the EM map. The homology model of the mutated BRCT domain fit well within the given density. Proximal to the BRCA1^5382insC^ homology model, there was a small region of unoccupied density that accommodated a single ubiquitin (pdbcode, 1UBQ)^23^ (Figure 5a, red). A similarly sized unoccupied density was protruding from the RNAP II core complex, near the mutated BRCA1–BARD1 RING domain. This density also accommodated a single ubiquitin (pdbcode, 1UBQ)^23^ (Figure 5a, orange).

To compliment our structural studies, and shed light on the nature of the ubiquitin moieties present in the mutated complexes, we performed co-IP experiments. Similar to the wild-type assemblies, BRCA1^5382insC^ interacted with phosphorylated RNAP II (Figure 5b). Also, the RNAP II large subunit contained K63-linked ubiquitin moieties, which is consistent with our structural findings. In contrast to wild-type BRCA1, we found that BRCA1^5382insC^ was the likely target of multiple K48-ubiqutin linkages as indicated by the smeared band present in western blots of the BRCA1 co-IPs (Figure 5c). This information suggested the extra density adjacent to the mutated BRCT domain was a potential linkage site for K48-specific ubiquitin moieties. Additional ubiquitin chains attached to BRCA1 may be flexible and hence not visible in our density map. These modifications suggested that the signal for DNA repair on the RNAP II core complex was conserved in the mutated assemblies, but that BRCA1^5382insC^ had acquired modifications to direct its degradation by the proteasome.

### The BRCA1^5382insC^ mutation alters protein interactions with BARD1

Biochemical experiments have shown that the BRCA1^5382insC^ mutation weakens native protein interactions in the nucleus.^27^ One important nuclear interaction affected by this mutation is the heterodimer formed by the BRCA1 and BARD1 RING domains. The results presented here suggest that BRCA1 may contain K48-linked ubiquitin groups proximal to the BRCA1^5382insC^ mutation. This region in the BRCA1 protein is located distal to the BARD1-binding site. How then, does a mutation in the BRCT domain affect protein–protein interactions that are primarily at the N-terminus of BRCA1?

Similar to BRCA1, BARD1 contains BRCT motifs at its C-terminus. These motifs in BARD1 are also known to bind to phosphorylated peptide substrates having a pS-X-X-pF consensus sequence.^27^ On the basis of this information, we predicted that the BRCT of BARD1 interacts with the central domain of BRCA1, known as the serine-containing domain (SCD). The SCD region of BRCA1 contains multiple sites for ubiqutination and phosphorylation. Therefore, reduced interactions between mutated BRCA1 and BARD1 may be influenced by the addition of ubiquitin moieties in the SCD region of BRCA1.

To test these ideas biochemically, we probed the enzymatic accessibility of the SCD region of wild type and mutated BRCA1 contained in the nuclear fractions of breast cancer cell lines. We used phosphatase assays to assess the extent by which wild type and mutant BRCA1 can be dephosphorylated. The same total protein concentration of nuclear material (10 μg) was incubated with either lamba phosphatase (40 μl, 16,000 units; New England Biolabs) or buffer solution lacking the enzyme as a negative control. The mixtures were then analyzed by SDS-polyacrylamide gel electrophoresis (PAGE) and western blot analysis.

Western blots of the phosphatase digests and control samples were probed with antibodies against BRCA1 (AB1; Millipore). We observed a bandshift in the nuclear extracts that contained wild-type BRCA1 and lamba phosphastase (Figure 5c, top right). Control mixtures lacking the enzyme did not show this bandshift. By comparison, we observed no bandshifts in the treated and untreated nuclear material containing BRCA1^5382insC^ (Figure 5c, bottom right). These results suggested that some of the phosphorylation sites in the BRCA1^5382insC^ protein were inaccessible to phosphatase cleavage. This information also supports the idea that protein misfolding in the mutated BRCA1^5382insC^ can lead to ubiquitination in the SCD, and possibly hinder the proper binding interactions with BARD1.

## Discussion

Here we present the first 3D comparison of wild type and mutated BRCA1 protein assemblies derived from human breast cancer cells. Employing the recently developed tunable microchip system, we could enrich for and selectively isolate BRCA1 nuclear assemblies while still maintaining native protein–protein interactions. We found that wild type and mutated BRCA1^5382insC^ interacted directly with the RNAP II core, which was modified with K63-type ubiquitin moieties. As this modification to the RNAP II core is a known signal for DNA repair,^20^ the structures of the BRCA1 complexes presented here are likely primed for this function. Differences between the wild type and mutated assemblies included altered positioning of the RING domains in the density maps, DNA-binding capacity, ubiquitination patterns, and biochemical interactions with BARD1.

These results also complement previous biochemical studies that demonstrate how other forms of ubiqutination can lead to the degradation of transcriptional assemblies.^28^ On the basis of our new molecular insights, we found that ubiquitination has an important role in protein complex formation during the early stages of transcription. Ongoing investigations aimed at understanding the structural complexity of BRCA1 assemblies during various stages of RNA synthesis will help to delineate the functional relevance of these interactions.

In a broader sense, our combined structural and biochemical approaches provide a unique opportunity to study native protein interactions related to both normal and diseased processes. On a technical front, the widespread use of commercially available protein adapters can enhance future microchip applications toward a variety of disease conditions. As such, our tunable approach may help shed light on the inner-workings of native proteins in a unique way that has not been fully explored in human cancer research.

## Materials and methods

### Nuclear extraction and fractionation procedures

HCC70 and HCC1937 lines of human breast cancer cells (ATCC) were grown until near confluence in a 5% CO_2_ environment at 37 °C in RPMI-1640 medium (Mediatech, Manassas, VA, USA) supplemented with 10% fetal bovine serum (Fisher Scientific, Hanover Park, IL, USA). The cells were collected into pellets by detaching them using trypsin-EDTA (Life Technologies, Carlsbad, CA, USA), quickly centrifuging (500 *g*, 5 min) them and then washing them with phosphate-buffered saline (PBS). The cells were lysed using the NE-PER extraction kit (Thermo Scientific, Miami, OK, USA) and the nuclear contents were collected. After dilution to ~1 mg/ml in HEPES buffer (20 mmol/l HEPES, 2 mmol/l MgCl_2_, pH 7.2) supplemented with 5 mmol/l imidazole and protease inhibitor cocktail (EDTA-free, Roche, Branchburg, NJ, USA), the extracts were incubated with pre-equilibrated Ni–NTA) agarose beads (Qiagen, Hilden, Germany) to enrich for phosphorylated RNAP II. After 1-h incubation at 4 °C on the clinical rotator, the solution was added to a column and the flow-through was collected and reserved for later analysis. The column was washed three times with HEPES buffer supplemented with 140 mmol/l NaCl and 5 mmol/l imidazole. The addition of HEPES buffer with NaCl supplemented with 150 mmol/l imidiazole resulted in the elution of proteins. The Bradford Assay (Thermo Scientific) was used to estimate all protein concentrations.

### Phosphatase assays

For phosphatase digestion, two tubes were prepared for each cell line (HCC70 & HCC1937) with one tube serving as the experimental tube and the other serving as the control sample. Approximately 10 μg of protein was loaded into each tube. The following buffers were added to the tubes (experimental & control): 10 μl of 10× MnCl_2_ (New England Biolabs, Ipswich, MA, USA), 10 μl of 10× PMP (New England Biolabs), 1 μl of 100× protease inhibitor cocktail (EDTA-free, Roche). Lambda phosphatase (40 μl; 16,000 units) was added to the experimental tube only while additional buffer solution was added to the control tube. Each tube was incubated in a water bath at 37 °C for 90 min. After the incubation, aliquots of the samples (3.25 μg of total protein) were prepared for western blot analysis. We used 3–8% NuPAGE Bis–Tris mini gels with Tris-Acetate running buffer to perform SDS-PAGE analysis. The separated proteins were transferred onto an Immobolin-P membrane (Millipore, Billerica, MA, USA) in a Mini-PROTEAN Tetra system (Bio-Rad, Hercules, CA, USA). Blocking solution (1% non-fat dry milk (NFDM)) was added to the blot for 1 h with gentle rocking. The blots were incubated overnight at 4 °C with primary antibody solution against the BRCA1 RING domain (Millipore, AB1, MS110) diluted to 0.001 mg/ml in 1% NFDM. After 3 washes with TBS-T solution (standard TBS containing 0.05% Tween-20 (Fisher, Hanover Park, IL, USA)), the blots were incubated for 1 h with HPRO-conjugated anti-mouse secondary antibody (Jackson ImmunoResearch, West Grove, PA, USA). Following the incubation step, the membranes were again washed 3 times with TBS-T. ECL Prime Western blotting reagent (GE Healthcare, Marlborough, MA, USA) was applied to the blot for detection using a ChemiDoc MP (Bio-Rad) for imaging purposes.

### Co-IP experiments

The eluates from the Ni–NTA agarose beads were supplemented with protease inhibitor and phosphatase inhibitor cocktail (Thermo Scientific). Antibody (5 μg) diluted in PBS-T (0.02% Tween-20, Fisher) was combined with 0.75 mg Dynabeads Protein G (Life Technologies) and incubated with rotation for 30 min at 4 °C. Antibodies used in the immunoprecipitation were POLR2C (Abcam, Cambridge, MA, USA, ab138436), BRCA1 (Santa Cruz Biotechnology, Dallas, TX, USA (SCBT) sc-642, C-20), BARD1 (SCBT sc-11438, H-300), RPL3 (Abcam ab83098) and normal mouse IgG (SCBT sc-2025). After the antibody-coated beads were washed with HEPES buffer, the eluates were added and immunoprecipitated overnight with gentle rotation at 4 °C. HEPES buffer was used to wash the beads (three times) and the proteins were eluted with NuPAGE LDS sample buffer. A 4–12% NuPAGE Bis–Tris mini gel with MOPS running buffer was used to separate the proteins. Following separation, the proteins were transferred onto an Immobilon-P membrane (Millipore) in a Mini-PROTEAN Tetra system (Bio-Rad). Blocking solution (1% NFDM or 4% bovine serum albumin (SCBT)) was added to blots with gentle rocking for 1 h. TBS-T was applied 3 consecutive times after incubation with blocking buffer to wash the blots. The blots were incubated with primary antibody, diluted in 1% NFDM or bovine serum albumin solution, overnight at 4 °C. Other antibodies used were RNAP II (SCBT sc-9001, H-224), RNA Polymerase II H5 and H14 (Covance, Raleigh, NC, USA, MMS-129 and MMS-134), Polyubiquitin (K63-linkage-specific, Enzo, Farmingdale, NY, USA, BML-PW0600) and ubiquitin (K48-linkage-specific, Abcam ab140601). After three washes with TBS-T (0.05% Tween-20), either goat anti-rabbit or goat anti-mouse secondary antibodies conjugated to horseradish peroxide (Jackson ImmunoResearch) were added to the blots and incubated for 1 h. A ChemiDoc MP (Bio-Rad) was used for imaging and ECL Prime western blotting reagent (GE Healthcare) for detection.

### Preparation of tunable microchip samples

Functionalized Ni–NTA (Avanti Polar Lipids, Alabaster, AL, USA) lipid monolayers were formed over 15-μl aliquots of Milli-Q water on parafilm and incubated for 1 h in a sealed petri dish. Negatively stained specimens required 5% Ni–NTA and cryo-EM specimens required 25% Ni–NTA. C-flat grids with 2-μm holes and 1-μm of spacer (2/1) between holes (Protochips, Morrisville, NC, USA) or Cryo-SiN microchips (TEMwindows, West Henrietta, NY, USA) were placed on the surface of each monolayer. Each grid or microchip was removed from the monolayer surface and incubated for 1 min with aliquots (3-μl) of His-tagged Protein A (0.01 mg/ml) (Abcam) in buffer solution containing 50 mM HEPES (pH 7.5), 150 mmol/l NaCl, 10 mmol/l MgCl_2_, and 10 mmol/l CaCl_2_. The protein-A coated chips were blotted to remove excess solution and 3-μl aliquots of IgG antibodies (0.01 mg/ml), in the same buffer as protein A, were added. Antibodies against the BRCA1 C-terminus (SCBT, sc-642, C-20) and the RING domain (Millipore, MS110, AB1) were employed for the BRCA1 labeling experiments. A Hamilton syringe was utilized to remove the antibody solution from the grid surface after a 1-min incubation. The protein fractions collected during the Ni–NTA chromatography step described above were incubated with the enhanced chips (Cryo-SiN)^10^ for 2 min. Following a wash with Milli-Q water, the grids were stained with 0.2% uranyl formate or plunge-frozen into liquid ethane using a Cryoplunge 3 device equipped with GentleBlot technology (Gatan, Pleasanton, CA, USA).

### Electron microscopy and image analysis

The BRCA1-associated transcriptional complexes were viewed with a FEI BioTwin Transmission Electron Microscope (FEI Company) equipped with a LaB_6_ filament at 120 kV under low-dose conditions (~5 electrons per Å^2^) for both negatively stained and cryo-EM samples. A FEI Eagle 2 k HS CCD camera (FEI Company) recorded the images with a pixel size of 30-μm at a nominal magnification of 50,000× (for wild-type complexes) and 68,000× (for mutant complexes), for a final sampling of 6 Å per pixel and 4.4 Å per pixel, respectively. The same TEM conditions were used to collect images of negatively stained and ice-embedded specimens except for varying the defocus range. Roughly 22,000 particles of wild-type complexes bound to DNA were selected from EM images by the automated program, PARTICLE^11^ and the selected particle images were exported to the RELION software package.^12^ The RELION software package was used to refine and reconstruct the individual complexes employing an initial model for the RNAP II core complex (pdbcode, 4A93)^18^ low-pass filtered to a resolution of 80 Å. The initial model was only used in the first round of the refinement to assign initial orientation parameters to each particle. Later iterations were heavily dependent on the experimental data to refine the assigned angles by setting the regularization parameter to *T*=4. We followed standard reconstruction routines and employed a pixel size of 6 Å to produce a final composite 3D structure masked at ~250 Å with a resolution filtered to 2.2 nm. The RELION software package identified variable structures present in our image stack. Five distinct structures were output by the RELION software package independent of the user-defined starting parameters based on Bayesian statistical comparisons computed between the initial model and the experimental particle images. The degrees of DNA occupancy varied among the five structures, each of which contained ~4,400 particles and we highlighted density maps having the lowest and the highest DNA occupancy. For comparison, we selected ~3,000 particles of mutant BRCA1^5382insC^ complexes, and performed the same reconstruction routines, but using a pixel size of 4.4 Å, as images were acquired at a nominal magnification of ×68,000. RELION identified one major class during refinement.

### Molecular modeling

The pSer5 motif found within the C-terminus of RNAP II was previously reported (pdbcode, 4H3K^23^). Using the Chimera software package,^29^ we found that the peptide fit within the binding cleft of the BRCT. The Chimera program established the most optimum fit by employing an energy minimization strategy. The energy minimization technique implemented algorithms to calculate the amount of force generated by different atom arrangements while assessing the atomic positions requiring the least amount of force. Chimera calculated the energy minimization method to find a local minimum without crossing energy barriers or searching for global minimums. This step is achieved by following an iterative optimization procedure where the force is calculated on each atom. Atoms are then moved by a computed step predicted to decrease the force. This process was iterated until the measured force falls below a set threshold. This strategy is useful in biochemical studies as the arrangement that generates the least amount of force correlates to the most likely arrangement present in nature.
