# Supplementary material for: A microchip platform for structural oncology applications
Source: NPJ Breast Cancer. 2016 Jun 15;2:16016–. doi: 10.1038/npjbcancer.2016.16 (PMC5003533; doi:10.1038/npjbcancer.2016.16)
Supplement: Supplementary Information [file npjbcancer201616-s1.pdf]

A microchip platform for structural oncology applications

Carly E. Winton[1,2], Brian L. Gilmore[1], Andrew C. Demmert[1,3], Zhi Sheng[1,3] and Deborah F. Kelly[1,2,3,4]

[1]Virginia Tech Carilion Research Institute, Roanoke, VA 24016, United States

[2]School of Biomedical Engineering and Science, Virginia Tech, Blacksburg, VA 24061, United States

[3]Department of Internal Medicine, Virginia Tech Carilion School of Medicine, Roanoke, VA 24016, United States

[4]Department of Biological Sciences, Virginia Tech, Blacksburg, VA 24061, United States

*Correspondence to: debkelly@vt.edu

Supplementary Information

Supplementary Figures

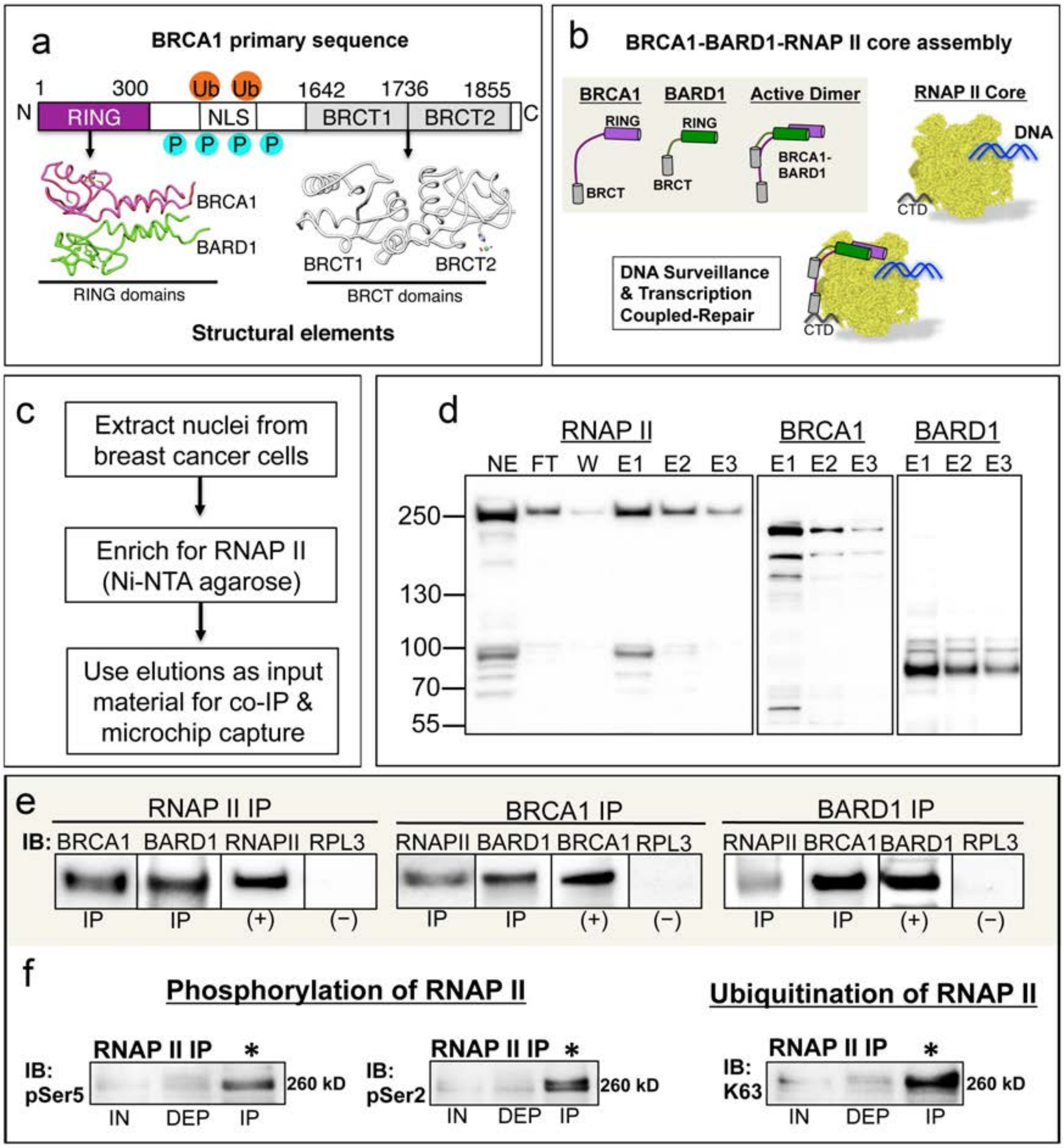

Supplementary Figure S1. BRCA1, RNAP II, and BARD1 interact in the enriched nuclear fractions of triple negative breast cancer cells. (a) The primary sequence and structural elements of BRCA1 include the N-terminal RING domain (magenta) that associates with BARD1 (green) (pdbcode, 1JM7).[1] The central region of BRCA1

contains a nuclear localization sequence (NLS) and sites for phosphorylation (P, cyan) and ubiquitination (Ub, orange). The C-terminus of BRCA1 is composed of tandem BRCT domains (gray, pdbcode, 1JNX).[2] **(b)** Schematic to describe how the BRCA1-BARD1 heterodimer may interact with the RNAP II core during DNA surveillance and transcriptional repair events. **(c)** Our strategy to enrich for native BRCA1-assoicated transcriptional complexes from triple negative breast cancer cells. **(d)** RNAP II, wild type BRCA1, and BARD1 co-elute (E1 – E3) from Ni-NTA agarose beads. **(e)** Co-IP experiments on the eluates revealed protein interactions along with positive (+) and negative (-) controls. (**f**) Western blot analysis of co-IP experiments showed the RNAP II core was phosphorylated at pSer5 and pSer2 peptide repeats (**left**) and contained K63-linked ubiquitin moieties (**right**). *Denotes IP results that indicate post-translational modifications. RPL3 (ribosomal large subunit 3); IN (input material); DEP (ubound material); IP (immunoprecipitated interaction); IB (immunoblot)

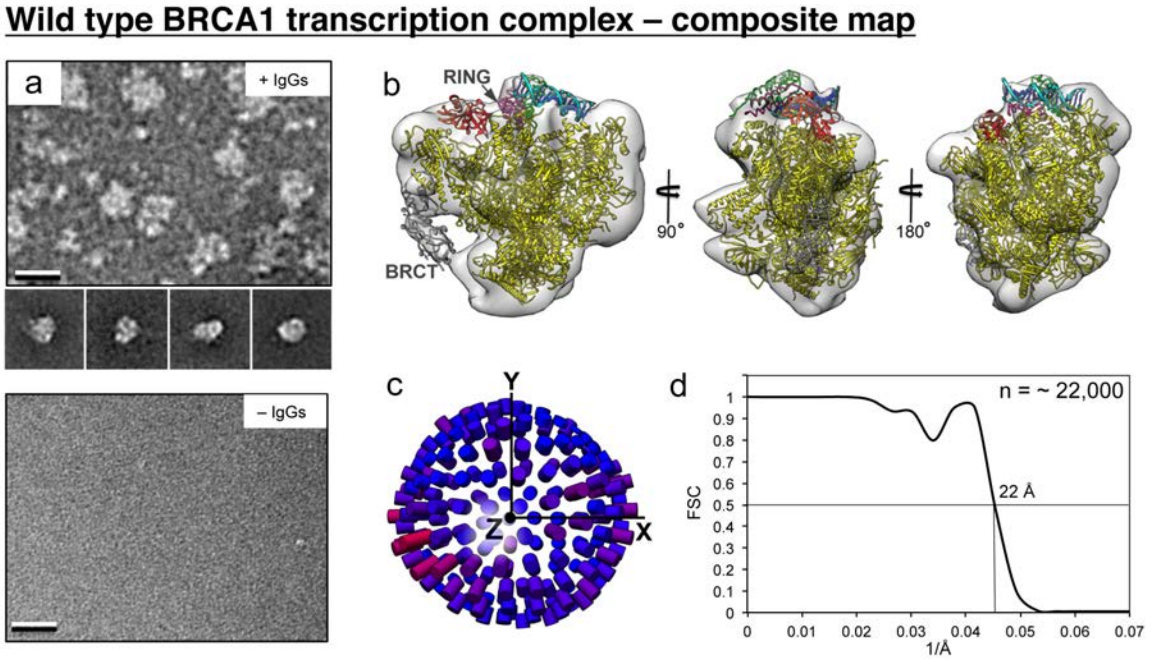

Supplementary Figure S2. EM analysis of the wild type BRCA1 transcription complex – composite map. (a) Representative image and class averages of wild type BRCA1 complexes tethered to the microchip surface using antibodies against BRCA1 (**+IgGs**, **top**). Microchips lacking antibodies **(–IgGs, bottom)** failed to capture appreciable quantities of protein complexes. Scale bar is 25 nm. Width of each panel of averages is 36 nm. **(b)** Different orientations of the wild type composite structure (please also see Figure 2). **(c)** Angular distribution plot of particle projections with color designations (blue to red) showing an increase in the number of particles at a given coordinate.[3] **(d)** Fourier Shell Correlation (FSC) curve to indicate a nominal resolution of ~22 Å for the composite structure comprised.[3]

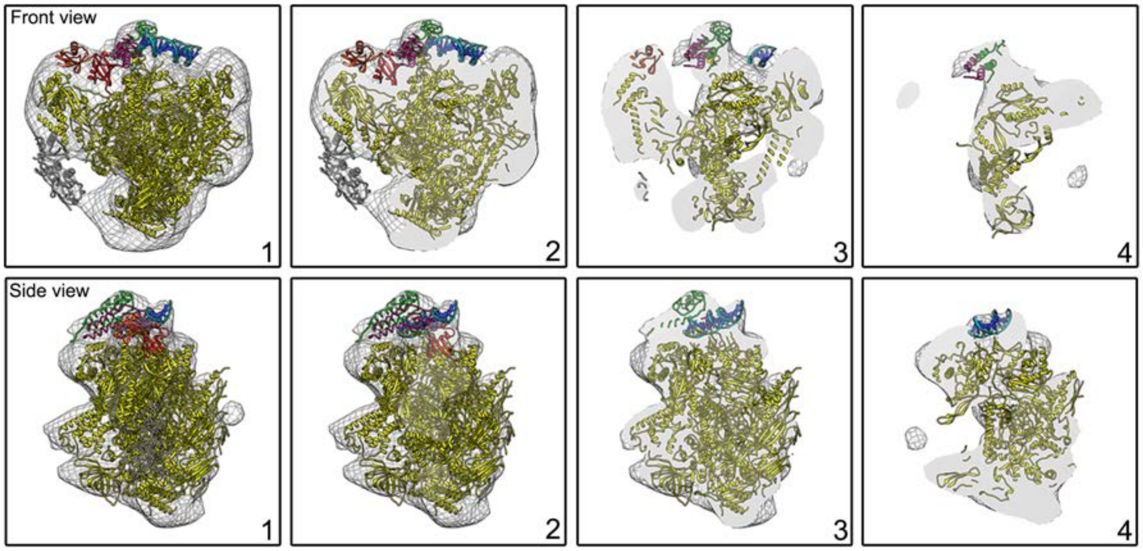

Supplementary Figure S3. Segments through the wild type composite reconstruction overlaid with atomic models. Representative slices (1 – 4) through the wild type 3D structure (orthogonal views) illustrate the placement of the BRCA1-BARD1 RING domains (pink and green; pdbcode, 1JM7),[1] the BRCT domain (gray; pdbcode, 1JNX),[2] a short strand of DNA (blue), the RNAP II core (yellow; pdbcode, 4A93),[4] and ubiquitin moieties (red, orange; pdbcode 1UBQ).[5] Please see **Supplementary Movie S1**.

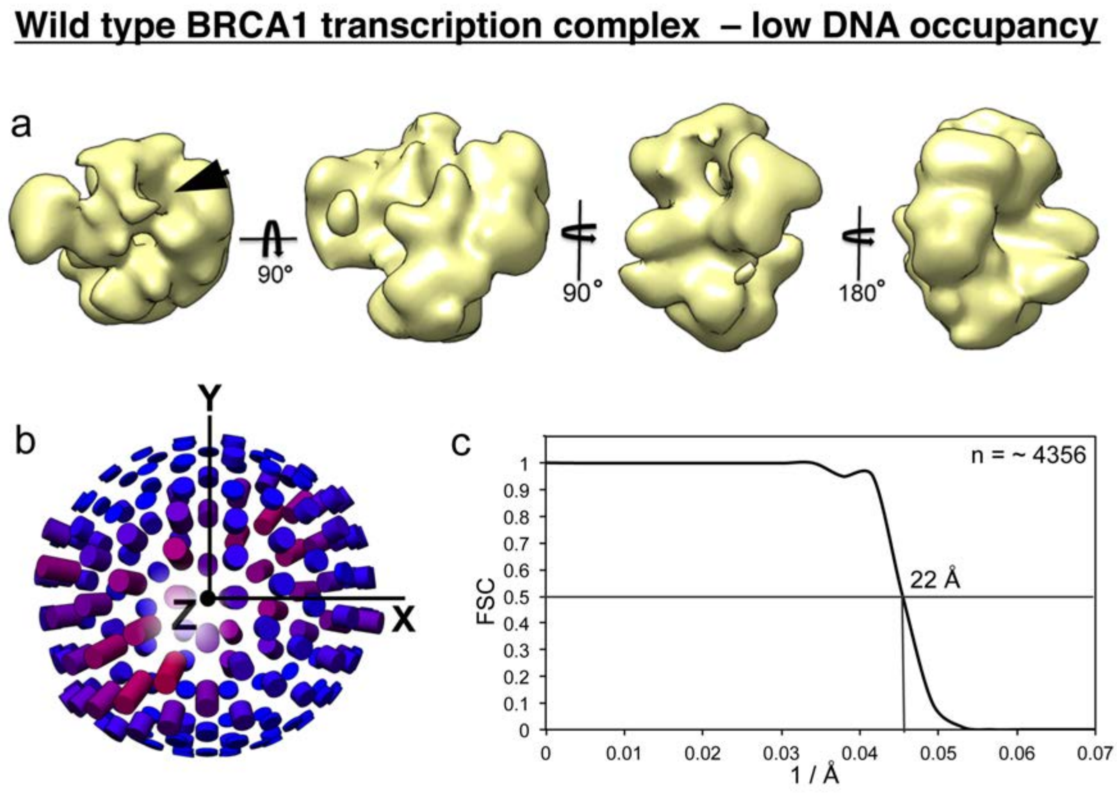

Supplementary Figure S4. EM analysis of the wild type BRCA1 transcription complex having low DNA occupancy. **(a)** Different orientations of the 3D structure having low DNA occupancy (please also see Figure 3). **(b)** Angular distribution plot of particle projections. **(c)** FSC curve to indicate a nominal resolution of ~22 Å for the reconstruction containing low DNA occupancy. Please see **Supplementary Movie S2**.

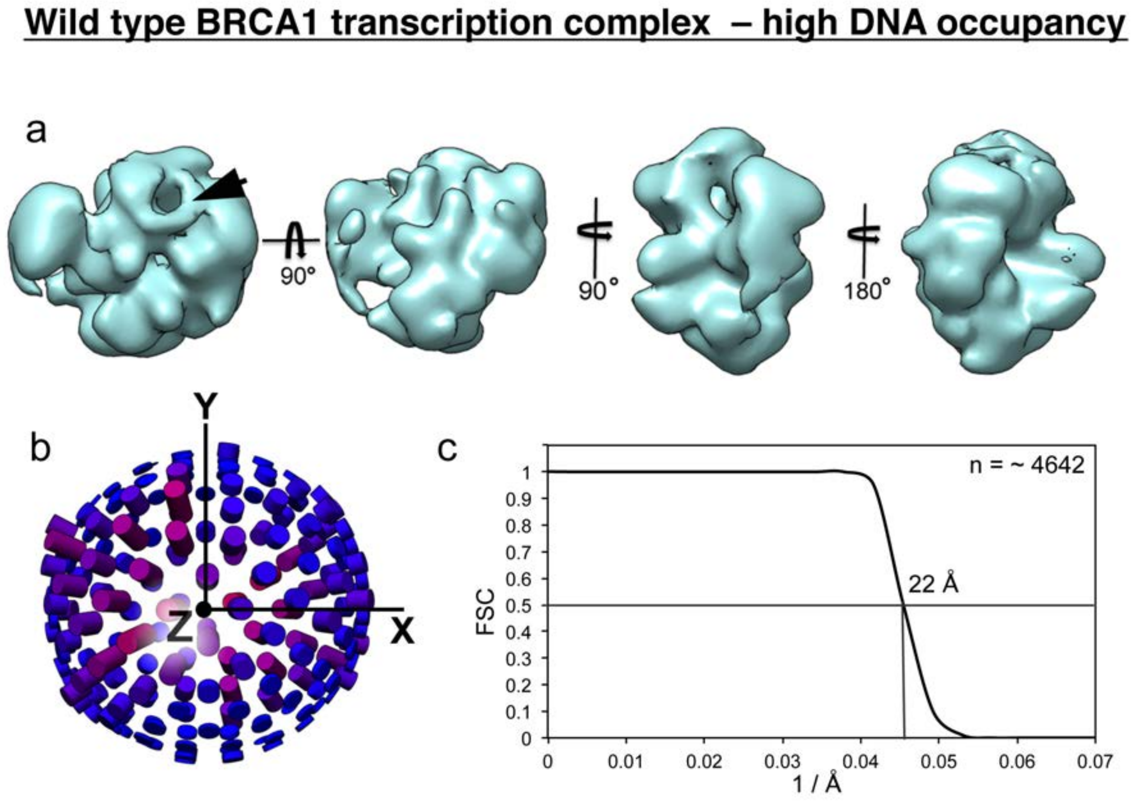

Supplementary Figure S5. EM analysis of the wild type BRCA1 transcription complex having high DNA occupancy. **(a)** Different orientations of the 3D structure having high DNA occupancy (please also see Figure 3). **(b)** Angular distribution plot of particle projections. **(c)** FSC curve to indicate a nominal resolution of ~22 Å for the reconstruction containing high DNA occupancy. Please see **Supplementary Movie S3**.

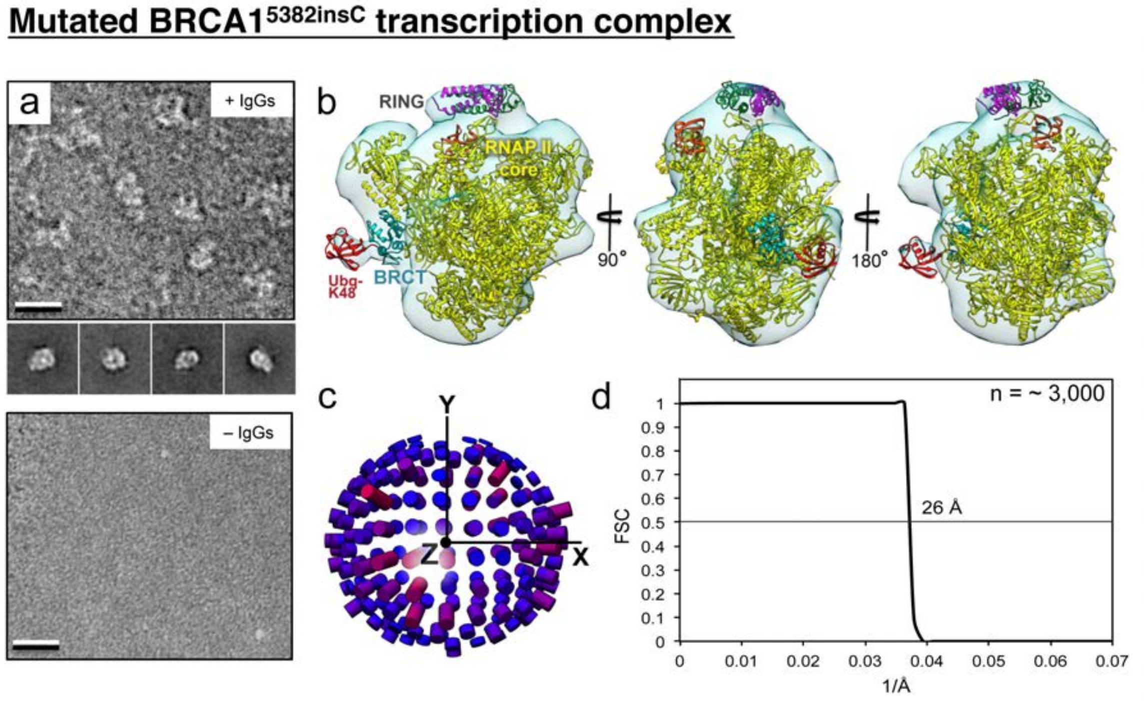

Supplementary Figure S6. EM analysis of the mutated BRCA1^{5382insC} transcription complex. **(a)** Representative image and class averages of mutated BRCA1^{5382insC} complexes tethered to the microchip surface using antibodies against BRCA1 (**+IgGs**, **top**). Microchips lacking antibodies **(–IgGs, bottom)** failed to capture appreciable quantities of protein complexes. Scale bar is 25 nm. Width of each panel of averages is 36 nm. **(b)** Different orientations of the mutated BRCA1^{5382insC} complex structure (please also see Figure 5). **(c)** Angular distribution plot of particle projections. **(d)** Fourier Shell Correlation (FSC) curve to indicate a nominal resolution of ~26 Å for the mutated BRCA1^{5382insC} complex.

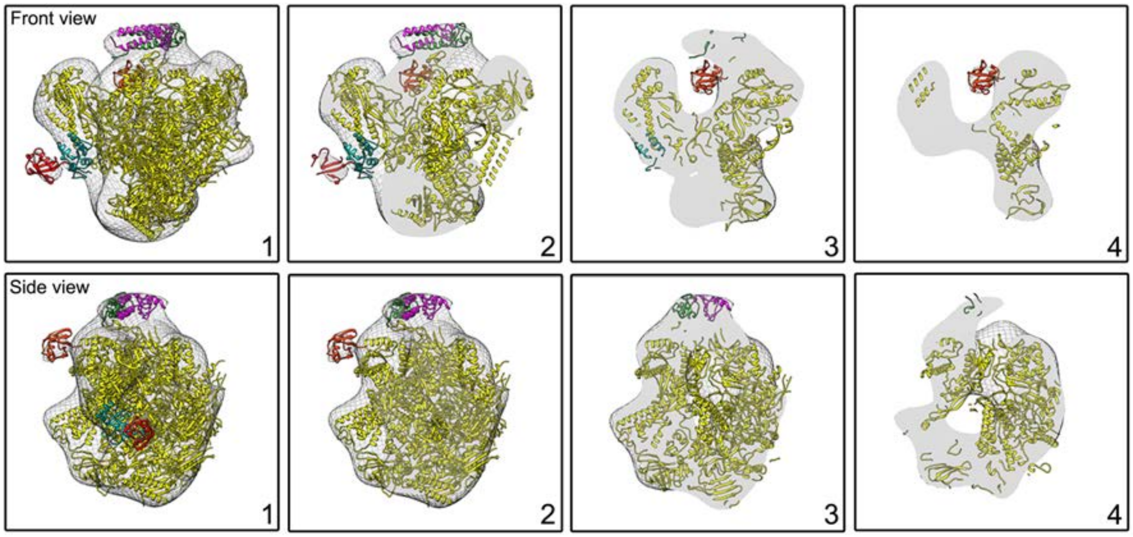

Supplementary Figure S7. Segments through the mutated BRCA1^{5382insC} reconstruction overlaid with atomic models. Representative slices (1 – 4) through the mutated BRCA1^{5382insC} complex structure (orthogonal views) illustrate the placement of the BRCA1-BARD1 RING domains (pink and green; pdbcode, 1JM7),[1] the BRCT domain (aqua, pdbcode, 1JNX),[2] the RNAP II core (yellow, pdbcode, 4A93),[4] and ubiquitin moieties (red, orange, pdbcode 1UBQ).[5] Please see **Supplementary Movie S5**.

Supplementary Movies

Supplementary Movie S1. Compiled slices through the wild type composite structure overlaid with atomic models. Sections through the wild type 3D structure overlaid with atomic models for the BRCA1-BARD1 RING domains (pink and green; pdbcode, 1JM7),[1] the BRCT domain (gray; pdbcode, 1JNX),[2] the RNAP II core (yellow; pdbcode, 4A93),[4] and ubiquitin moieties (red, orange; pdbcode 1UBQ).[5] This movie accompanies Supplementary Figure S3.

Supplementary Movie S2. Transient intermediate structures illustrate variability with respect to the low DNA occupancy map. The low DNA occupancy model (yellow) was transitioned to the high DNA occupancy model (cyan) using the morph conformation function in the Chimera software package.[6] The atomic models for BRCA1-BARD1 (pink and green; pdbcode, 1JM7),[1] DNA (blue), and two ubiquitin moieties (red; pdbcode, 1UBQ)[5] were overlaid with respect the low occupancy model. A short strand of DNA fits within the DNA binding region.

Supplementary Movie S3. Transient intermediate structures illustrate variability with respect to the high DNA occupancy map. The low DNA occupancy model (yellow) was transitioned to the high DNA occupancy model (cyan) using the morph conformation function in the Chimera software package.[6] The atomic models for BRCA1-BARD1 (gray; pdbcode 1JM7),[1] DNA (blue), and two ubiquitin moieties (red; pdbcode 1UBQ)[4] were overlaid with respect the high occupancy model. A larger strand of DNA fits within the DNA binding region.

Supplementary Movie S4. Molecular model of the pSer5 peptide fit within the wild type BRCT binding pocket. Employing molecular overlay functions in the Chimera software package,[6] the pSer5 peptide (pSPSY) (pdbcode, 4H3K)[7] was superimposed onto the pSPTF peptide (cyan) determined in the BRCT structure (gray; pdbcode, 3K0H).[8]

Supplementary Movie S5. Compiled slices through the mutated BRCA1^{5382insC} complex structure overlaid with atomic models. Sections through the mutated BRCA1^{5382insC} complex structure overlaid with atomic models for the BRCA1-BARD1 RING domains (pink and green; pdbcode, 1JM7),[1] the BRCT domain (aqua; pdbcode, 1JNX),[2] the RNAP II core (yellow; pdbcode, 4A93),[4] and ubiquitin moieties (red, orange; pdbcode 1UBQ).[5] This movie accompanies Supplementary Figure S7.

<u>**References:**</u>

1 Brzovic PS, Rajagopal P, Hoyt DW, King MC, Klevit RE. Structure of a BRCA1-BARD1 heterodimeric RING-RING complex. *Nat Struct Biol* 2001; **8**: 833-837.

2 Williams RS, Green R, Glover JN. Crystal structure of the BRCT repeat region from the breast cancer-associated protein BRCA1. *Nat Struct Biol* 2001; **8**: 838-842.

3 Gilmore BL, Winton CE, Demmert AC, Tanner JR, Bowman S, Karageorge V, Patel K, Sheng Z, Kelly DF. A Molecular Toolkit to Visualize Native Protein Assemblies in the Context of Human Disease. *Sci Rep* 2015; **5**: 14440.

4 Walmacq C, Cheung AC, Kireeva ML, Lubkowska L, Ye C, Gotte D *et al*. Mechanism of translesion transcription by RNA polymerase II and its role in cellular resistance to DNA damage. *Mol Cell* 2012; **46**: 18-29.

5 Vijay-Kumar S, Bugg CE, Cook WJ. Structure of ubiquitin refined at 1.8 A resolution. *Journal of molecular biology* 1987; **194**: 531-544.

6 Pettersen EF, Goddard TD, Huang CC, Couch GS, Greenblatt DM, Meng EC *et al*. UCSF Chimera--a visualization system for exploratory research and analysis. *J Comput Chem* 2004; **25**: 1605-1612.

7 Xiang K, Manley JL, Tong L. An unexpected binding mode for a Pol II CTD peptide phosphorylated at Ser7 in the active site of the CTD phosphatase Ssu72. *Genes Dev* 2012; **26**: 2265-2270.

8 Campbell SJ, Edwards RA, Glover JN. Comparison of the structures and peptide binding specificities of the BRCT domains of MDC1 and BRCA1. *Structure* 2010; **18**: 167-176.
